# Better Night’s Sleep and Subjective Cognition: The Role of Day and Night Work Shifts

**DOI:** 10.1093/geroni/igaa057.1372

**Published:** 2020-12-16

**Authors:** Britney Veal, Christina Mu, Soomi Lee

**Affiliations:** University of South Florida, Tampa, Florida, United States

## Abstract

Previous research indicates poor sleep and cognitive functioning are associated. Studies have yet to consider the role of work shift on this relationship. The current study examined the sleep and subjective cognition relationship in nurses, and if this relationship differed for day- and night- shift nurses. Sixty-one nurses (M=35.39, SD=11.73; 39 day-, 22 night-shift) reported their nightly sleep characteristics and next-day subjective cognition (i.e., processing speed, memory, and mental focus) using ecological momentary assessments for 2 weeks. Multilevel models controlled for sociodemographic characteristics and decomposed the variance attributed by between- and within-person levels. At the within-person level, better sleep the previous night was associated with better subjective cognition the following day. This relationship was more apparent in night-shift nurses than in day-shift nurses, such that (a) longer sleep duration predicted better mental focus (B=1.62, p<.05) and (b) higher sleep quality predicted better memory (B=8.67, p<.001). At the between-person level, better sleep overall was associated with better subjective cognition across days. This association was more apparent in day-shift nurses than in night-shift nurses, such that (a) better sleep quality and sufficiency predicted faster processing speed (B=34.33; B=26.28; p<.001) and (b) better sleep quality and greater sleep sufficiency predicted better memory (B=30.94; B=23.09; p<.001). Findings suggest that sleep characteristics are associated with subjective cognition in nurses day-to-day and on average. Specific sleep characteristics associated with subjective cognition differ between day- and night-shift nurses, presumably due to differences in their sleep issues and perceived cognitive abilities.

